# By using machine learning and *in vitro* testing, SERPINH1 functions as a novel tumorigenic and immunogenic gene and predicts immunotherapy response in osteosarcoma

**DOI:** 10.3389/fonc.2023.1180191

**Published:** 2023-04-05

**Authors:** Guang Xia, Song Wu, Ke Luo, Xiaoyu Cui

**Affiliations:** ^1^ Department of Orthopaedics, Third Xiangya Hospital, Central South University, Changsha, China; ^2^ Department of Anesthesiology, Hunan Cancer Hospital, Central South University, Changsha, Hunan, China; ^3^ Department of Anesthesiology, Third Xiangya Hospital, Central South University, Changsha, China

**Keywords:** SERPINH1, tumor microenvironment, immunotherapy, osteosarcoma, machine learning

## Abstract

**Introduction:**

The most prevalent bone tumor with a relatively high level of aggressiveness and malignancy is osteosarcoma. The characteristics of the serpin family in osteosarcoma have not been defined.

**Methods:**

In this study, the predictive significance of the serpin superfamily was investigated in the osteosarcoma and Gene Expression Omnibus (GEO) databases from The Cancer Genome Atlas (TCGA).

**Results:**

It was discovered that SERPINH1 is a significant biological marker in osteosarcoma. According to the CCK-8, EdU, and Transwell assays as well as the IHC assay, SERPINH1 may promote osteosarcoma proliferation and migration. It is also more expressed in tumor samples than in healthy samples. SERPINH1 might forecast the effects of immunotherapy. Additionally, immune cells are interacted with through checkpoint, cytokine, and growth factor pathways in osteosarcomas with high SERPINH1 levels. The biological function, immunological characteristics, and treatment response (immunotherapy and chemotherapy responses) of patients with osteosarcoma were successfully predicted using a model related to SERPINH1. SERPINH1 and the SERPINH1-related score predict ferroptosis/pyroptosis/apoptosis/necroptosis in osteosarcoma.

**Discussion:**

The SERPINH1-related score was an effective method for identifying osteosarcoma patients who would respond to immunotherapy and chemotherapy, as well as for predicting the survival outcomes of such patients.

## Introduction

The most prevalent bone tumor with a relatively high level of aggressiveness and malignancy is osteosarcoma. Children and teenagers are frequently affected by osteosarcoma, which has an annual incidence of 1 to 3 incidences per million persons globally (1). Osteosarcoma typically involves lung metastasis and mostly develops from primitive mesenchymal cells found in bone. Surgery to remove pathogenic lesions and adjuvant chemotherapy are still the go-to treatments for people with osteosarcoma (2). Sometimes, the cure rates are underwhelming. The scientific community has faced a formidable challenge in enhancing the chances of survival for people with osteosarcoma (3). A thorough understanding of osteosarcoma biology has been made possible by the rapid advancement of high-throughput sequencing, growing viability of molecular profiling, and reliable model systems based on extensive bioinformatics research.

A popular family of protease inhibitors called serpin uses conformational changes to block the activity of the target enzyme. The coagulation route, inflammation, immunology, and cancer are just a few of the crucial proteolytic cascades that the serpin superfamily regulates (4). It has been demonstrated that the brain metastasis Serpin superfamily promotes tumor growth and vascular co-option (5). A biomarker for colorectal cancer that interacts with CEA was identified as SERPINB5 (6). The motility and invasiveness of oral carcinoma cells may be aided by overexpressed SERPINB1 (7). Notably, SERPINE2 could encourage osteosarcoma tumor cell proliferation and medication resistance (8). The serpin superfamily in osteosarcoma has not yet been thoroughly examined, yet. It is still unknown if the serpin superfamily has a significant predictive significance in osteosarcoma. As SERPINH1 has been widely studied in pan-cancer except for osteosarcoma (9, 10), the prognostic value of SERPINH1 in osteosarcoma was reasonably expected. We, therefore, paid special attention to SERPINH1 and performed a comprehensive analysis on its role in osteosarcoma.

The prognostic utility of the serpin superfamily was investigated in this study. It was discovered that SERPINH1 is a significant biological marker in osteosarcoma. The physical function, immunological characteristics, and medication response of patients with osteosarcoma were successfully predicted using a SERPINH1-related model.

## Method

### Data gathering

The TCGA osteosarcoma and GSE21257 databases were used to gather the osteosarcoma samples. The data were transformed to TPM value. The R package SVA was used for data standardization and batch-to-batch difference removal.

### Value for prognosis of SERPINH1

Based on the cutpoint value of SERPINH1 determined using the R package survminer, the osteosarcoma patients were split into two groups. The R package pROC was used to create the time-dependent receiver operating characteristic (ROC) curve for SERPINH1. On SERPINH1, the gene set variation analysis (GSVA) of the Kyoto Encyclopedia of Genes and Genomes (KEGG) and gene ontology (GO) pathways was carried out. The TISCH2 database was used to investigate the SERPINH1 expression pattern in the osteosarcoma tumor microenvironment (TME).

### Immunotherapy prediction of SERPINH1

The TIDE was used for the immunotherapy prediction of SERPINH1 (11). The TISMO was used for immunotherapy and cytokine treatment prediction of SERPINH1 (12).

### Immunohistochemistry (IHC)

Detailed methods were provided in the **Supplementary File** following the previous study (13).

### Cell culture

Detailed methods were provided in the **Supplementary File** following the previous study (13).

### Small interfering RNA (siRNA) transfection

The siRNAs sequences are as follows: SERPINH1-1 (F: GCAGCAAGCAGCACUACAATT R: UUGUAGUGCUGCUUGCUGCTT), SERPINH1-2 (F: CCAGCCUCAUCAUCCUCAUTT R: AUGAGGAUGAUGAGGCUGGTT), SERPINH1-3 (F: GGCCUAAGGGUGACAAGAUTT R: AUCUUGUCACCCUUAGGCCTT). The exact method is provided in the **Supplementary Material**.

### Real-time quantitative polymerase chain reaction (RT-qPCR)

The primer sequences are SERPINH1 (F: ATATTTATAGCCAGGTACCTTCTCACC R: TTTTATAGTTGGGAGAGGTTGGGATAG), GAPDH (F: AATGGGCAGCCGTTAGGAAA R: GCCCAATACGACCAAATCAGAG). The exact method is provided in the **Supplementary Material**.

### Cell counting Kit-8 (CCK-8) assay

The U2OS and MNNG/HOS cells were seeded into 96-well plates at 5,000 cells/well density. After 24h, 1/10 volume of CCK-8 reagent (Proteintech, USA) was added to the wells, and the absorbance value was detected at 450nm after one h incubation at 37°C. The exact method is provided in the **Supplementary Material**.

### EdU assay

Detailed methods were provided in the **Supplementary File** following the previous study (13).

### Transwell assay

The migration of U2OS and MNNG/HOS cells was assessed using a Transwell chamber (Corning, USA) with polycarbonic membranes (6.5 mm in diameter and eight μm pore size). Cells in serum-free medium were added into the upper chamber at the density of 5 × 10^5^ cells/ml (200 μl/well), and the culture medium with 10% FBS was added to the lower chamber. After incubating for 48h at 37°C, U2OS and MNNG/HOS cells that penetrated the lower surface were stained with 0.1% crystal violet and counted. The exact method is provided in the **Supplementary Material**.

SERPINH1 was the subject of a weighted correlation network analysis (WGCNA) in the TCGA dataset. The input was the matrix from the TCGA dataset. For further investigation, the genes from the turquoise module were taken out.

### Building the SERPINH1-related score

Between two SERPINH1-related groups, the differentially expressed genes (DEGs) were identified. The prognostic DEGs were discovered using a single-variable Cox regression analysis. Dimension reduction and the creation of the SERPINH1-related score employed the Random Survival Forest method and the least absolute shrinkage and selection operator (LASSO) technique. The expression value of the gene’s * coefficient was used to construct the SERPINH1-related score. The R package survival was used to create the Kaplan-Meier survival curve for the SERPINH1-related score. The R package pROC was used to create the time-dependent ROC curve for the SERPINH1-related score. The predicted clinical variables were identified using univariate and multivariate Cox regression analysis.

### Immune characteristics of the SERPINH1-related score

The correlation between the SERPINH1-related score and immune infiltrating cells (10 cells from MCPcounter algorithm (14), 28 cells from the ssGSEA algorithm (15), six cells from TIMER algorithm (16)) were analyzed. The association between the SERPINH1-related and microenvironment scores (17) (ESTIMATE, Immune, and Stromal) was investigated.

### Single-cell RNA sequencing (scRNA-seq) analysis on SERPINH1

For the scRNA-seq investigation, GSE152048 was employed. The R package “Seurat” was used to annotate the cell type. The R package “iTalk” was used to evaluate the cell communication pattern. The R package “monocle” was used to diagnose the pseudotime trajectory analysis.

### Immunotherapy prediction of the SERPINH1-related score

The correlation between the SERPINH1-related score and classical immune checkpoints was analyzed. The association between the SERPINH1-related score and APM (antigen processing and presenting machinery) score, Cytotoxic activity (CYT), T cell-inflamed gene expression profile (GEP), and interferon γ (IFN-γ) were analyzed (18–20). The TIDE was used for immunotherapy prediction of the SERPINH1-related score (11).

### Drug prediction using the SERPINH1-related score

The chemotherapy medications associated with the SERPINH1-related score were predicted using the R package OncoPredict (21).

## Results

### Prognostic value of serpin superfamily and SERPINH1

**Figure 1:**
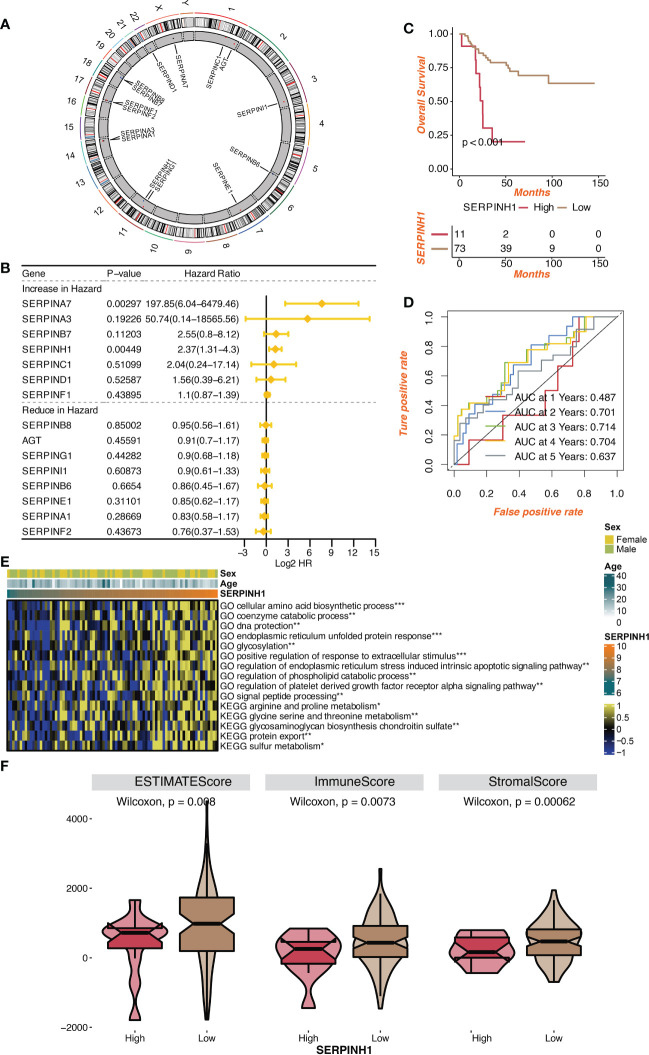
Prognostic value of serpin superfamily and SERPINH1. **(A)** Circplot of the serpin superfamily in human chromosomes. **(B)** Univariate Cox regression analysis on the serpin superfamily. **(C)** Survival curves of the two SERPINH1-stratified groups. **(D)** The 1-year, 2-year, 3-year, 4-year, and 5-year ROC regarding SERPINH1. **(E)** The correlation between SERPINH1 and GO, KEGG terms quantified by GSVA. **(F)** The levels of microenvironment scores in two SERPINH1 groups. *P<0.05; **P<0.01; ***P<0.001.

**Figure 1A** depicts the location of the serpin superfamily on human chromosomes. In patients with osteosarcoma, SERPINH1 and SERPINA7 were found to be two independent prognostic indicators by univariate Cox regression analysis on the serpin superfamily (**Figure 1B**). We focused specifically on SERPINH1, which has drawn more attention from the scientific community since it is more likely to contribute to the development of osteosarcoma as a malignancy. Patients with osteosarcoma were divided into two groups according to the expression level of SERPINH1, and it was shown that the two groups had dramatically different survival rates (**Figure 1C**). The prognostic value of SERPINH1 was validated by the 1-year, 2-year, 3-year, 4-year, and 5-year ROCs, which have the respective values of 0.487, 0.701, 0.714, 0.704, and 0.637. (**Figure 1D**). SERPINH1 was favorably related with biological processes, including cellular amino acid biosynthetic process, glycosylation, phospholipid carbolic process control, and protein export, according to GSVA of GO and KEGG keywords (**Figure 1E**). In the low SERPINH1 group, microenvironment scores such as ESTIMATE, Immune, and Stromal scores were significantly higher (**Figure 1F**).

### Immunotherapy prediction of SERPINH1

**Figure 2:**
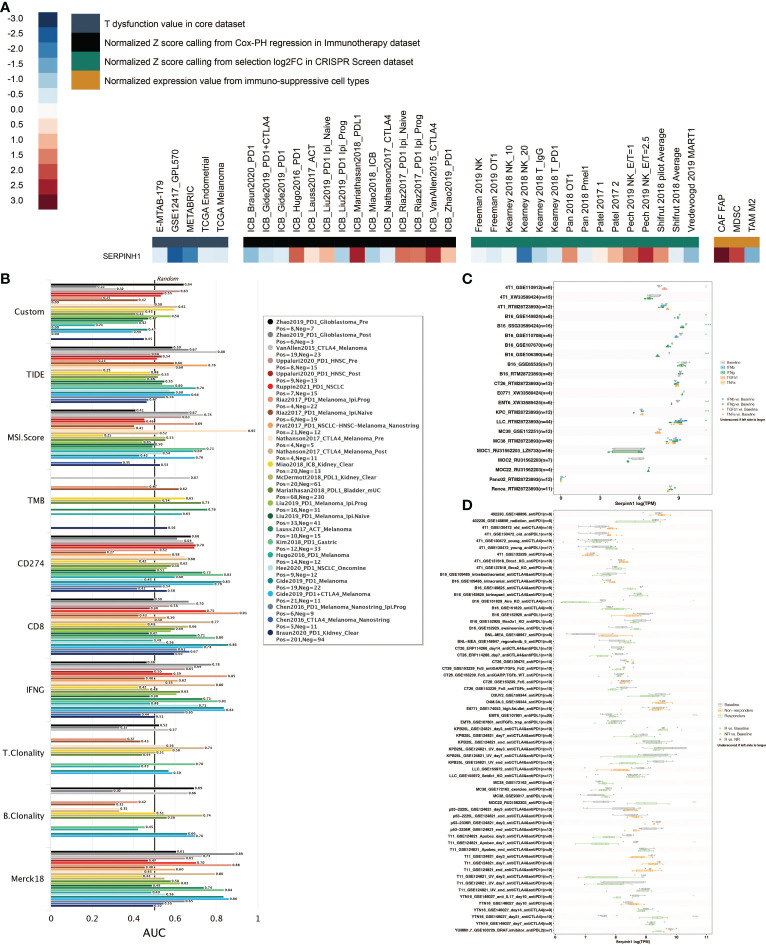
Immunotherapy prediction of SERPINH1. **(A)** The regulator prioritization module in TIDE. **(B)** Comparison of SERPINH1 and immunotherapy determinants in human immunotherapy datasets. **(C)** The prediction of SERPINH1 in cytokine treatment (IFN-β, IFN-γ, TGF-β, and TNF-α) in murine datasets. **(D)** The prediction of SERPINH1 in immunotherapy in murine datasets. *P<0.05; **P<0.01; ***P<0.001.

SERPINH1 was given the highest priority possible for mechanistic follow-up research using TIDE’s regulator prioritization module (**Figure 2A**). Based on the Normalized Z score calling using Cox-PH regression in the Immunotherapy dataset, high expression of SERPINH1 was discovered in the ICB Mariathasan2018 PDL1 and ICB VanAllen2015 CTLA4 datasets. Based on the Normalized Z score calling from selection log2FC in the CRISPR Screen dataset, it was discovered that SERPINH1 had a high expression level in the Pech 2019 NK E/T=2.5 dataset. Based on normalized expression values from immunosuppressive cell types, increased expression of SERPINH1 was discovered in CAF FAP and MDSC. Eight human immunotherapy datasets that included SERPINH1 achieved AUC values greater than 0.5. (**Figure 2B**). SERPINH1 exhibited a higher predictive value than TMB and B. Clonality, which respectively gave AUC values of > 0.5 in seven and seven immunotherapy cohorts. The predictive value of SERPINH1 was, however, lower than the MSI score (AUC > 0.5 in 13 immunotherapy cohorts), T.Clonality (AUC > 0.5 in 9 immunotherapy cohorts), CD274 (AUC > 0.5 in 21 immunotherapy cohorts), TIDE (AUC > 0.5 in 18 immunotherapy cohorts), IFNG (AUC > 0.5 in 17 immunotherapy cohorts), and CD8 (AUC > 0.5 in 18 immunotherapy cohorts). In 11 mouse datasets, SERPINH1 could accurately predict the effects of cytokine treatment (IFN, IFN, TGF, and TNF) (**Figure 2C**). SERPINH1 might accurately forecast immunotherapy in 5 murine datasets (**Figure 2D**).

**Figure 3:**
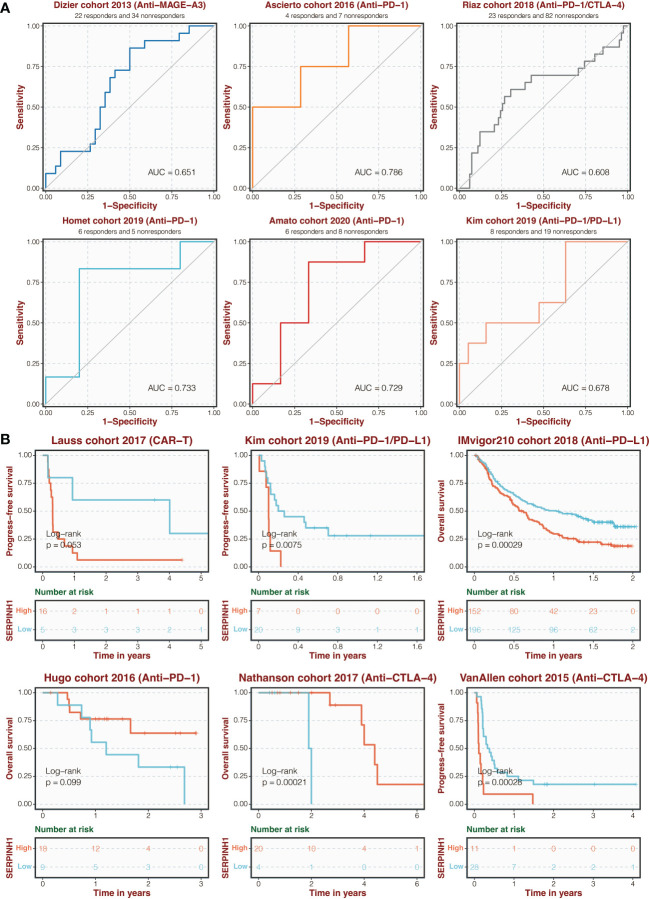
Immunotherapy prediction of SERPINH1. **(A)** ROC regarding the sensitivity of SERPINH1 in immunotherapy prediction in six immunotherapy cohorts. **(B)** Survival curves of the two SERPINH1-stratified groups in six immunotherapy cohorts.

Additionally, in six immunotherapy cohorts, including Dizier (AUC = 0.651), Ascierto (AUC = 0.786), Riaz (AUC = 0.608), Homet (AUC = 0.733), Amato (AUC = 0.729), and Kim (AUC = 0.678), SERPINH1 shown excellent sensitivity in predicting immunotherapy response (**Figure 3A**). Patients in Hugo and Nathanson cohorts had more remarkable survival outcomes with high SERPINH1 expression, while patients in Lauss, Kim, IMvigor210, and Van Allen cohorts had more remarkable survival outcomes with low SERPINH1 expression (**Figure 3B**).

### Pan-cancer analysis on the serpin superfamily

Pan-cancer mutation analysis on the serpin superfamily was performed. The expression pattern of the serpin superfamily between tumor and normal samples is shown in **Figure S1A**. The methylation difference of the serpin superfamily between tumor and normal samples is shown in **Figure S1B**. The heterozygous CNV of the serpin superfamily is shown in **Figure S1C**. The homozygous CNV of the serpin superfamily is shown in **Figure S1D**. Pan-cancer function analysis on the serpin superfamily was performed. Pathway annotation on the serpin superfamily is shown in **Figure S2A**, in which EMT activation showed a strong positive correlation with the serpin superfamily. miRNA network annotation on the serpin superfamily is shown in **Figure S2B**. Pan-cancer drug prediction analysis on the serpin superfamily was performed based on the CTRP database (**Figure S3A**) and the GDSC database (**Figure S3B**), in which SERPINB6, SERPINE1, and SERPINH1 could predict most of the commonly used chemotherapy drugs. We further explored the disease network of SERPINH1, in which SERPINH1 was highly enriched in osteogenesis processes such as osteogenesis imperfecta (**Figure S4A**). Besides, the protein interaction network of SERPINH1 showed that HSPA8, MIA3, COL1A1, COL1A2, COL1A4, COL26A1, FKBP10, PPIB, LEPRE1, and CRTAP highly connected with SERPINH1 (**Figure S4B**).

### *In vitro* validation on SERPINH1

**Figure 4:**
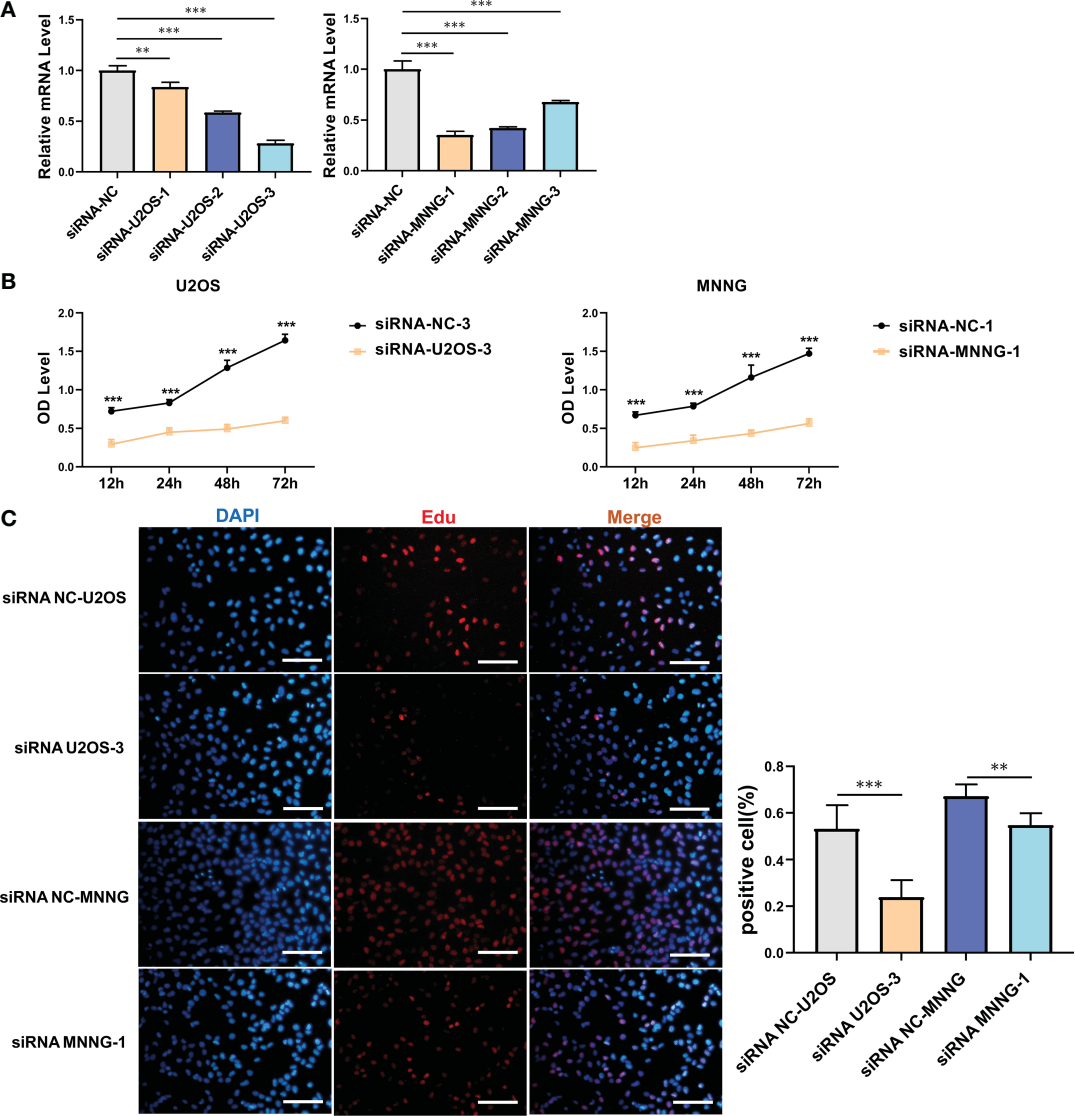
*In vitro* validation on SERPINH1. **(A)** RT-qPCR results of the expression of SERPINH1 in four groups (si-NC, si-SERPINH1-1, si-SERPINH1-2, si-SERPINH1-3) in two cell lines. **(B)** CCK-8 assay in two groups (si-NC, si-SERPINH1). In two cell lines, **(C)** EdU assay in two groups (si-NC, si-SERPINH1). **P<0.01; ***P<0.001.

**Figure 5:**
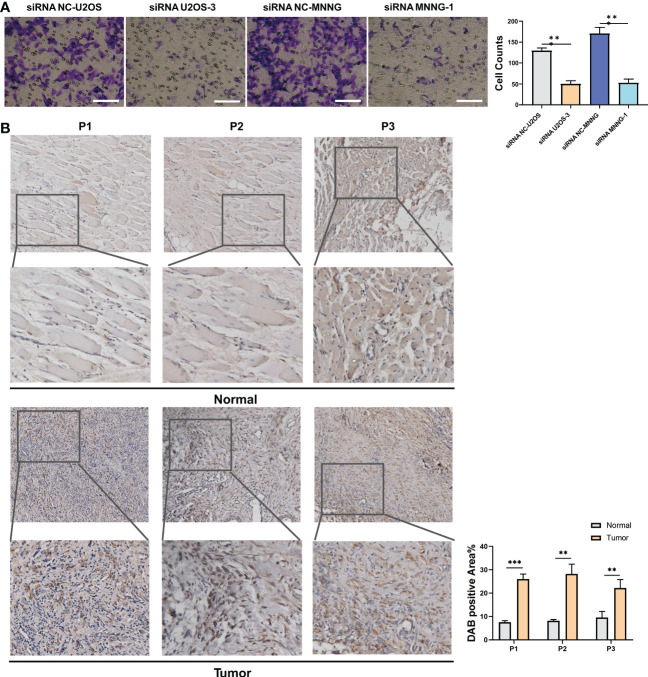
*In vitro* validation on SERPINH1. **(A)** Transwell assay in two groups (si-NC, si-SERPINH1) in two cell lines. **(B)** IHC results of the expression of SERPINH1 in osteosarcoma tumor tissues and normal tissues. **P<0.01; ***P<0.001.

It was investigated how SERPINH1 functions biologically in osteosarcoma. The expression of SERPINH1 was considerably reduced in three si-SERPINH1 groups compared to the si-NC group in U2OS and MNNG/HOS cells, according to the results of RT-qPCR (**Figure 4A**). For the follow-up experiment, si-SERPINH1, which has the most critical ability to suppress the expression of SERPINH1, was utilized. CCK-8 (**Figure 4B**) and EdU tests (**Figure 4C**) demonstrated that infection with si-SERPINH1 greatly reduced the ability of U2OS and MNNG/HOS cells to proliferate. Transwell experiment revealed that after exposure to si-SERPINH1, U2OS and MNNG/HOS cells’ ability to migrate was drastically reduced (**Figure 5A**). The IHC results further demonstrated that osteosarcoma tumor tissues had much greater levels of SERPINH1 expression than did normal tissues (**Figure 5B**).

### Construction of the SERPINH1-related score

**Figure 6:**
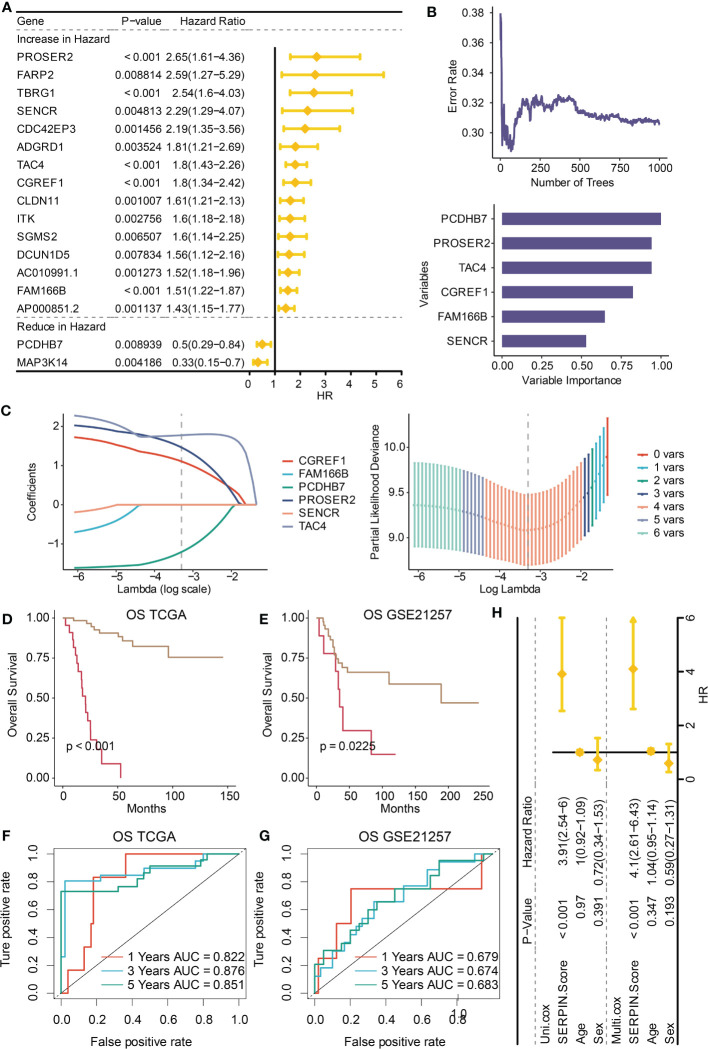
Construction of the SERPINH1-related score. **(A)** Univariate Cox regression analysis on the prognostic DEGs. **(B)** Machine learning Random Survival Forest for dimension reduction. **(C)** Machine learning LASSO for dimension reduction and constructing the SERPINH1-related score. **(D)** Survival curves of the two SERPINH1-related score-stratified groups in the TCGA dataset. **(E)** Survival curves of the two SERPINH1-related score-stratified groups in the GSE21257 dataset. **(F)** The 1-year, 3-year, and 5-year ROC regarding the SERPINH1-related score in the TCGA dataset. **(G)** The 1-year, 3-year, and 5-year ROC regarding the SERPINH1-related score in the GSE21257 dataset. **(H)** Univariate and multivariate Cox regression analysis on the clinical factors.

**Figure 7:**
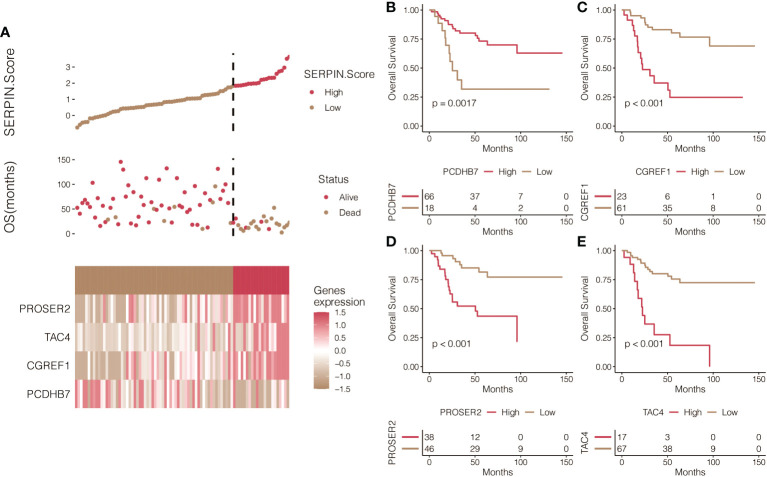
Prognostic value of the SERPINH1-related score. **(A)** The expression patterns of PCDHB7, TAC4, PROSER2, and CGREF1 in two SERPINH1-related score-stratified groups. **(B)** Survival curves of the two PCDHB7-stratified, **(C)** TAC4-stratified, **(D)** PROSER2-stratified, and **(E)** CGREF1-stratified groups.

It was determined that 17 DEGs were predictive genes, of which 15 were risky indicators and two were beneficial markers (**Figure 6A**). Using machine learning Random Survival Forest, the most effective prognostic genes were chosen (**Figure 6B**). Additionally, the most potent predictor genes were identified using machine learning LASSO, and the SERPINH1-related score was created (**Figure 6C**). The following formula was used to determine the SERPINH1 score: 1.1077*CGREF1, 1.7743*TAC4, 1.4629*PROSER2, and -1.2053*PCDHB7. SERPINH1-related score was used to classify osteosarcoma patients. **Figure 7A** displays the expression profiles of PCDHB7, TAC4, PROSER2, and CGREF1 in two score-stratified groups associated to SERPINH1. Osteosarcoma patients with higher PCDHB7 expression exhibited better survival rates (**Figure 7B**), while those with lower CGREF1, PROSER2, and TAC4 expression had worse survival rates (**Figures 7C, D, E**). The two SERPINH1-related score-stratified groups in the TCGA and GSE21257 datasets had significantly different survival outcomes (**Figures 6D, E**). In the TCGA dataset, the 1-year, 3-year, and 5-year ROC values for the SERPINH1-related score are 0.822, 0.876, and 0.851, respectively (**Figure 6F**). In contrast, the GSE21257 dataset’s 1-year, 3-year, and 5-year ROCs for the SERPINH1-related score had the respective values of 0.679, 0.674, and 0.683 (**Figure 6G**), confirming the predictive usefulness of the SERPINH1-related score. The SERPINH1-related score was shown to be an independent predictive factor by univariate and multivariate Cox regression analysis on the clinical variables (**Figure 6H**).

### Immune characteristics of the SERPINH1-related score

**Figure 8:**
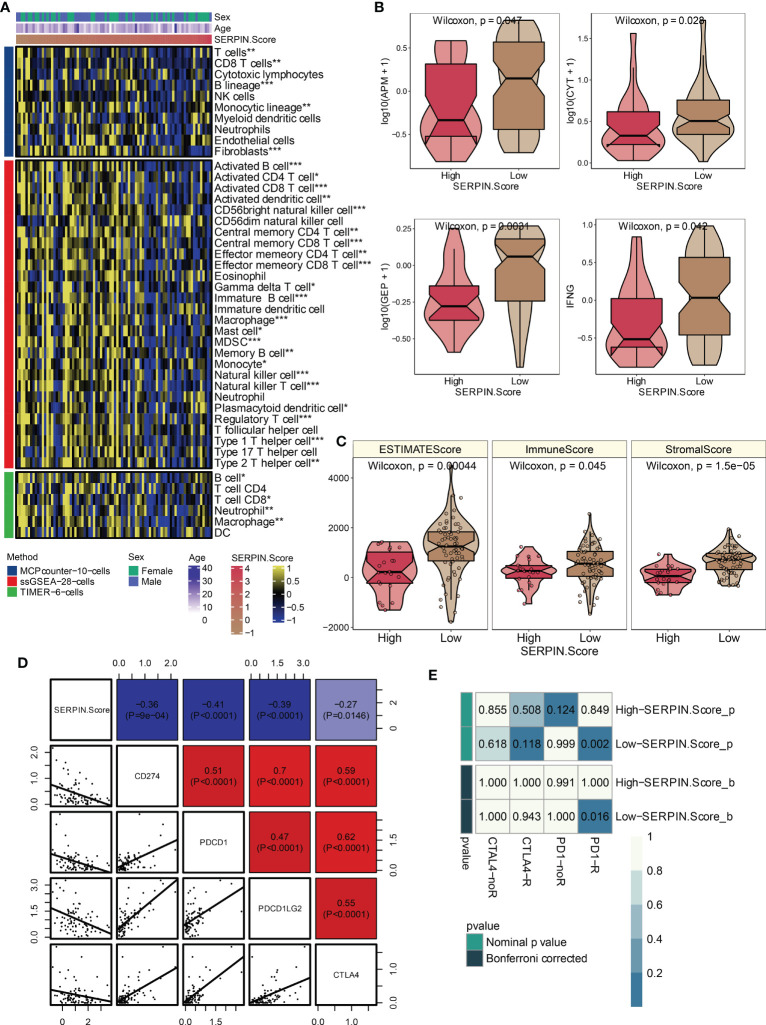
Immune characteristics of the SERPINH1-related score. **(A)** The correlation between the SERPINH1-related score and immune infiltrating cells. **(B)** APM, CYT, GEP, and IFN-γ in the two SERPINH1-related score-stratified groups. **(C)** ESTIMATE, Immune, and Stromal scores in the two SERPINH1-related score-stratified groups. **(D)** The correlation between the SERPINH1-related score and immune checkpoints. **(E)** TIDE-based immunotherapy prediction of the SERPINH1-related score. *P<0.05; **P<0.01; ***P<0.001.

Multiple immune infiltrating cells, including T cells, B cells, NK cells, macrophages, mast cells, Type 1 T helper cells, and Type 2 T helper cells, were observed to strongly negatively correlate with the SERPINH1-related score (**Figure 8A**). According to the aforementioned research, immune-cold microenvironment was present in osteosarcoma patients with high SERPINH1-related scores. In addition, the group with a high SERPINH1-related score exhibited considerably lower levels of the crucial immunotherapy determinants APM, CYT, GEP, and IFN- (**Figure 8B**). ESTIMATE, Immune, and Stromal scores were considerably lower in the group with high SERPINH1-related scores (**Figure 8C**). Positive correlations between the SERPINH1-related score and the traditional immunological checkpoints CD274, PD-1, PDCD1LG2, and CTLA-4 were observed (**Figure 8D**). Most importantly, patients with osteosarcoma who had low SERPINH1-related scores had a higher likelihood of responding to anti-PD-1 immunotherapy (**Figure 8E**).

### Biological functions of the SERPINH1-related score

**Figure 9:**
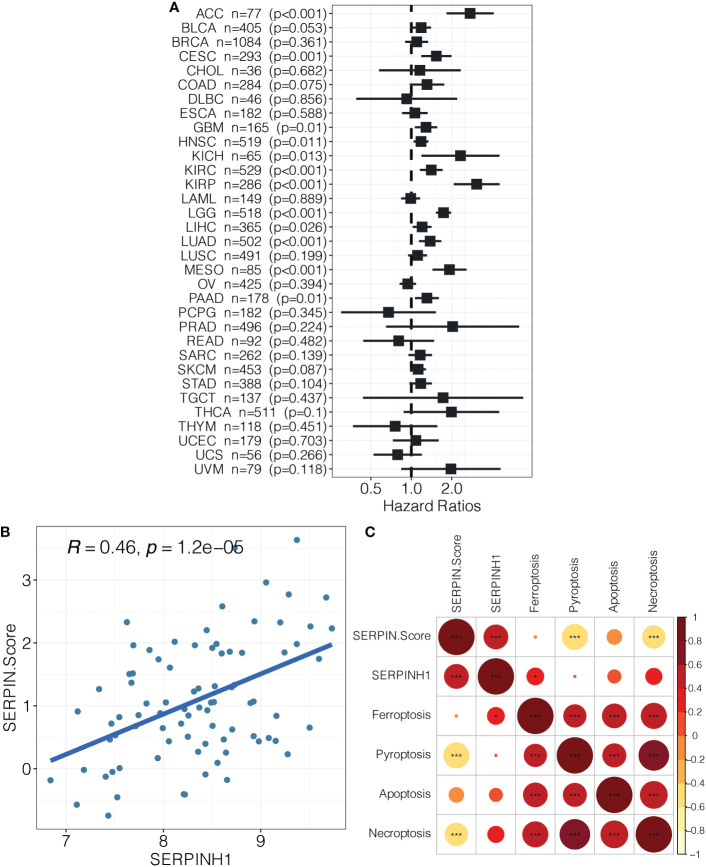
**(A)** Pan-cancer analysis on SERPINH1. **(B)** The correlation between SERPINH1 and SERPINH1-related score. **(C)** The correlation between SERPINH1, SERPINH1-related score, ferroptosis, pyroptosis, apoptosis, and necroptosis.

SERPINH1 was a hazardous marker in most of the cancer types (**Figure 9A**). Besides, SERPINH1 positively correlated with the SERPINH1-related score (**Figure 9B**). SERPINH1 positively correlated with ferroptosis, pyroptosis, apoptosis, and necroptosis (**Figure 9C**). Besides, the SERPINH1-related score positively correlated with ferroptosis, pyroptosis, apoptosis, and necroptosis (**Figure 9C**).

### Drug prediction of the SERPINH1-related score

**Figure 10:**
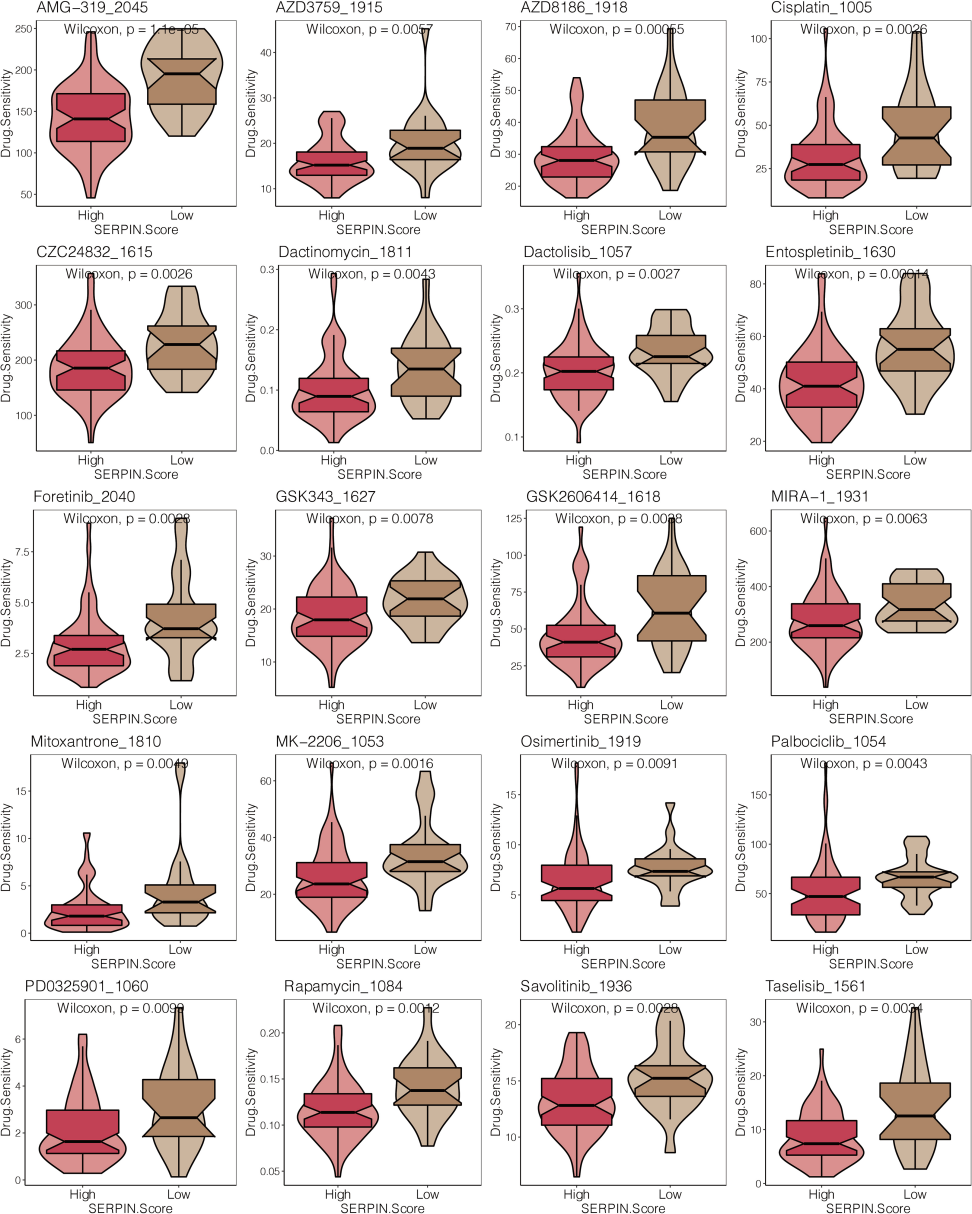
Drug prediction of the SERPINH1-related score. The drug sensitivity of 23 chemotherapy drugs in the two SERPINH1-related score-stratified groups.

Patients with osteosarcoma who had high SERPINH1-related scores had significantly lower drug sensitivity to AMG-319 2045, AZD3759 1915, AZD8186 1918, Cisplatin 1005, CZC24832 1615, Dactinomycin 1811, Dactolisib 1057, Entospletinib 1630, Foretinib 2040, GSK343 (**Figure 10**).

## Discussion

The most prevalent malignant bone tumor with a significant capacity for invasion and metastasis is osteosarcoma. Patients with osteosarcoma are currently treated mostly with surgery, radiation, chemotherapy, and neoadjuvant chemotherapy. But overall survival rates for osteosarcoma patients are still woefully inadequate. Additionally, some osteosarcoma patients are resistant to conventional chemotherapy medicines. Clinically speaking, treating people with osteosarcoma is quite difficult. Some modern treatments, including as antiangiogenic drugs, immunotherapies, and tumor apoptotic promoters, have been used to treat osteosarcoma, however it is still unknown how well they work. In order to screen the essential molecules or biomarkers for early diagnosis, targeted therapy, and prognosis analysis of osteosarcoma, it is crucial to have a complete understanding of the molecular pathological pathways relating to the occurrence and development of osteosarcoma.

Serpins are homologous proteins with different functions, including tumor development, blood coagulation, fibrinolysis, programmed cell death, and inflammation (22). The relationship between the serpin superfamily and the etiology of various malignancies has been the subject of a plethora of research. The specific roles of the serpin superfamily in osteosarcoma haven’t been explored in any studies, nevertheless. SERPINA7, SERPINA3, SERPINB7, SERPINH1, SERPINC1, SERPIND1, SERPINF1, SERPINB8, AGT, SERPING1, SERPINI1, SERPINB6, SERPINE1, SERPINA1, and SERPINF2 are the primary family members of the serpin superfamily. In this study, SERPINH1 outperformed other members of the serpin superfamily in terms of prognostic value. Specifically, SERPINH1 could regulate EMT and metastasis of gastric cancer *via* the Wnt/β-catenin signaling pathway (23). Splicing factor-derived circular RNA circCAMSAP1 was reported to accelerate the tumorigenic process of nasopharyngeal carcinoma *via* a SERPINH1/c-Myc positive feedback loop (24). SERPINH1 was found to be a potential prognostic biomarker based on a pan-cancer analysis (25). Circular RNA circ-TNPO3 could inhibit metastasis of clear cell renal cell carcinoma by binding to IGF2BP2 and destabilizing SERPINH1 (26). CyPA could interact with SERPINH1 to promote extracellular matrix production and inhibit the EMT of trophoblast (27). It has been established that SERPINH1 is crucial in controlling how biological processes in osteosarcoma are carried out. A potential target in the study of immunosuppressive TME and immunotherapy may also be SERPINH1. In human and mouse immunotherapy datasets, SERPINH1 displayed impressive performance in predicting cytokine treatment and immunotherapy.

Given the potent prognostic and predictive value of SERPINH1, we constructed the SERPINH1-related score based on the SERPINH1-related prognostic genes using two machine learning algorithms. PCDHB7, TAC4, PROSER2, and CGREF1 were finally included in the score. PCDHB7 was a potential marker in colorectal cancer (28). TAC4 was a potential marker in osteosarcoma (29). PROSER2 was a potential marker in melanoma (30). CGREF1 was a potential marker in prostate cancer (31). As expected, the SERPINH1-related score efficiently stratified the survival outcomes of osteosarcoma patients and served as an independent prognostic factor. Notably, SERPINH1 and the SERPINH1-related score predict ferroptosis/pyroptosis/apoptosis/necroptosis in osteosarcoma. In combating medication resistance, numerous preclinical and clinical trials have been conducted. Interesting correlations have been found between ferroptosis and cancer therapeutic resistance, and it has been shown that activating ferroptosis can overcome medication resistance (32). Recently, some studies found that pyroptosis can influence tumors’ proliferation, invasion, and metastasis, which is regulated by some non-coding RNAs and other molecules (33). Apoptosis is a coordinated and organized cellular process that takes place under both healthy and unhealthy circumstances. Moreover, it is one of the subjects that cell biologists study the most. One condition where insufficient apoptosis occurs is cancer, which results in malignant cells that resist death. Apoptosis has a complicated mechanism that encompasses numerous routes. Anywhere along these pathways, flaws might develop, resulting in the malignant transformation of the afflicted cells, tumor spread, and medication resistance. Despite being the root of the issue, apoptosis is a crucial component of many therapeutic regimens for cancer and plays a significant part in its management. The wealth of literature implies that it is possible to target apoptosis in cancer (34). Receptor-Interacting Protein 1 (RIP1), RIP3, and Mixed Lineage Kinase Domain-Like are the major mediators of necroptosis, a controlled necrotic cell death mode that is caspase-independent (MLKL). Necroptosis is a type of programmed cell death that can be used to treat cancer patients resistant to apoptosis. It can also activate and intensify antitumor immunity (35).

In recent years, researchers have learned more and more about the tumor microenvironment (36, 37). The tumor microenvironment consists of cellular components, including immune cells, endothelial cells, and fibroblasts, and non-cellular components, including extracellular matrix, cytokines, and hormones. Immune infiltrating cells play an essential role in influencing tumor progression and therapeutic response (38). In this study, osteosarcoma patients with high SERPINH1-related scores had lower immune infiltrating cells, including as T cells, B cells, NK cells, macrophages, mast cells, Type 1 T helper cells, and Type 2 T helper cells. In osteosarcoma patients with high SERPINH1-related scores, an immune-cold microenvironment that promotes tumor development may exist.

Immunotherapy has emerged as a promising treatment option for several cancers. Cancer immunotherapy tries to provide people immunity to fight cancer. In recent years, a large number of novel cancer-specific immunotherapeutic medicines have been approved, highlighting the effectiveness and promise of immunotherapy as an anticancer strategy (39). Among the multiple immunotherapy approaches, immune checkpoint inhibitor based on T cells has received the most attention due to their outstanding performance (40). APM, CYT, GEP, and IFN- levels were all statistically lower in the group with a high SERPINH1-related score, which were all important factors in immunotherapy. Additionally, the SERPINH1-related score was strongly adversely correlated with the CD274, PD-1, PDCD1LG2, and CTLA-4 immunological checkpoints. The likelihood of patients with osteosarcoma responding to anti-PD-1 immunotherapy was significantly higher in those with low SERPINH1-related scores. The SERPINH1-related score was accurate at identifying osteosarcoma patients who could respond to immunotherapy. Additionally, 23 chemotherapeutic agents had significantly decreased drug sensitivity in osteosarcoma patients with high SERPINH1-related scores. AZD3759 could inhibit the proliferation and progression of osteosarcoma through the blockade of the EGFR and JAK pathways (41). Inhibiting PI3Kβ with AZD8186 could regulate key metabolic pathways in PTEN-null tumors (42). Cisplatin is a well-known chemotherapeutic drug used to treat numerous human cancers, including bladder, head and neck, lung, ovarian, and testicular cancers (43). Therefore, the SERPINH1-related score was accurate in identifying osteosarcoma patients who could respond to chemotherapy.

## Conclusion

In conclusion, the biological role of SERPINH1 in osteosarcoma was investigated using *in vitro* confirmation. SERPINH1 and the SERPINH1-related score predict ferroptosis/pyroptosis/apoptosis/necroptosis in osteosarcoma. Further research is still needed to determine the precise processes underlying the osteosarcoma pathogenesis mediated by SERPINH1. The SERPINH1-related score was an effective method for identifying osteosarcoma patients who would respond to immunotherapy and chemotherapy, as well as for predicting the survival outcomes of such patients. A real-world cohort must be used to further validate the SERPINH1-related score’s universality.

## Data availability statement

The original contributions presented in the study are included in the article/**Supplementary Material**. Further inquiries can be directed to the corresponding author.

## Ethics statement

The studies involving human participants were reviewed and approved by institutional review board (IRB) of the Third Xiangya Hospital, Central South University. The patients/participants provided their written informed consent to participate in this study. Written informed consent was obtained from the individual (s) for the publication of any potentially identifiable images or data included in this article.

## Author contributions

GX conceived and performed most of the experiments. GX wrote the manuscript. SW and KL collected and analyzed the data. SW acquisition of the financial support for the project. XC provided experimental advice and supervised the study. All authors contributed to the article and approved the submitted version.

## References

[1] Kansara M Teng MW Smyth MJ Thomas DM. Translational biology of osteosarcoma. Nat Rev Cancer (2014) 14(11):722–35. doi: 10.1038/nrc3838 25319867

[2] Gill J Gorlick R. Advancing therapy for osteosarcoma. Nat Rev Clin Oncol (2021) 18(10):609–24. doi: 10.1038/s41571-021-00519-8 34131316

[3] Ritter J Bielack SS. Osteosarcoma. Ann Oncol (2010) 21 Suppl 7:vii320–325. doi: 10.1093/annonc/mdq276 20943636

[4] Heit C Jackson BC McAndrews M Wright MW Thompson DC Silverman GA. Update of the human and mouse SERPIN gene superfamily. Hum Genomics (2013) 7:22. doi: 10.1186/1479-7364-7-22 24172014 PMC3880077

[5] Valiente M Obenauf AC Jin X Chen Q Zhang XH Lee DJ. Serpins promote cancer cell survival and vascular co-option in brain metastasis. Cell (2014) 156(5):1002–16. doi: 10.1016/j.cell.2014.01.040 PMC3988473 24581498

[6] Baek JY Yeo HY Chang HJ Kim KH Kim SY Park JW. Serpin B5 is a CEA-interacting biomarker for colorectal cancer. Int J Cancer (2014) 134(7):1595–604. doi: 10.1002/ijc.28494 24114705

[7] Tseng MY Liu SY Chen HR Wu YJ Chiu CC Chan PT. Serine protease inhibitor (SERPIN) B1 promotes oral cancer cell motility and is over-expressed in invasive oral squamous cell carcinoma. Oral Oncol (2009) 45(9):771–6. doi: 10.1016/j.oraloncology.2008.11.013 19213596

[8] Mao M Wang W. SerpinE2 promotes multiple cell proliferation and drug resistance in osteosarcoma. Mol Med Rep (2016) 14(1):881–7. doi: 10.3892/mmr.2016.5316 27221371

[9] Zhong H Wang Z Wei X Liu Y Huang X Mo X. Prognostic and immunological role of SERPINH1 in pan-cancer. Front Genet (2022) 13:900495. doi: 10.3389/fgene.2022.900495 36105106 PMC9465257

[10] Zhang H Yan X Gu H Xue Q Liu X. High SERPINH1 expression predicts poor prognosis in lung adenocarcinoma. J Thorac Dis (2022) 14(12):4785–802. doi: 10.21037/jtd-22-1518 PMC9840017 36647484

[11] Jiang P Gu S Pan D Fu J Sahu A Hu X. Signatures of T cell dysfunction and exclusion predict cancer immunotherapy response. Nat Med (2018) 24(10):1550–8. doi: 10.1038/s41591-018-0136-1 PMC6487502 30127393

[12] Zeng Z Wong CJ Yang L Ouardaoui N Li D Zhang W. TISMO: syngeneic mouse tumor database to model tumor immunity and immunotherapy response. Nucleic Acids Res (2022) 50(D1):D1391–7. doi: 10.1093/nar/gkab804 PMC8728303 34534350

[13] Zhang L Wu S Huang J Shi Y Yin Y Cao X. A mitochondria-related signature for predicting immune microenvironment and therapeutic response in osteosarcoma. Front Oncol (2022) 12:1085065. doi: 10.3389/fonc.2022.1085065 36531021 PMC9751795

[14] Becht E Giraldo NA Lacroix L Buttard B Elarouci N Petitprez F. Estimating the population abundance of tissue-infiltrating immune and stromal cell populations using gene expression. Genome Biol (2016) 17(1):218. doi: 10.1186/s13059-016-1070-5 27765066 PMC5073889

[15] Charoentong P Finotello F Angelova M Mayer C Efremova M Rieder D. Pan-cancer immunogenomic analyses reveal genotype-immunophenotype relationships and predictors of response to checkpoint blockade. Cell Rep (2017) 18(1):248–62. doi: 10.1016/j.celrep.2016.12.019 28052254

[16] Li T Fan J Wang B Traugh N Chen Q Liu JS. TIMER: A web server for comprehensive analysis of tumor-infiltrating immune cells. Cancer Res (2017) 77(21):e108–10. doi: 10.1158/0008-5472.CAN-17-0307 PMC6042652 29092952

[17] Yoshihara K Shahmoradgoli M Martinez E Vegesna R Kim H Torres-Garcia W. Inferring tumour purity and stromal and immune cell admixture from expression data. Nat Commun (2013) 4:2612. doi: 10.1038/ncomms3612 24113773 PMC3826632

[18] Senbabaoglu Y Gejman RS Winer AG Liu M Van Allen EM de Velasco G. Tumor immune microenvironment characterization in clear cell renal cell carcinoma identifies prognostic and immunotherapeutically relevant messenger RNA signatures. Genome Biol (2016) 17(1):231. doi: 10.1186/s13059-016-1092-z 27855702 PMC5114739

[19] Ayers M Lunceford J Nebozhyn M Murphy E Loboda A Kaufman DR. IFN-gamma-related mRNA profile predicts clinical response to PD-1 blockade. J Clin Invest (2017) 127(8):2930–40. doi: 10.1172/JCI91190 PMC5531419 28650338

[20] Roh W Chen PL Reuben A Spencer CN Prieto PA Miller JP. Integrated molecular analysis of tumor biopsies on sequential CTLA-4 and PD-1 blockade reveals markers of response and resistance. Sci Transl Med (2017) 9(379):eaah3560. doi: 10.1126/scitranslmed.aah3560 28251903 PMC5819607

[21] Maeser D Gruener RF Huang RS. oncoPredict: an r package for predicting *in vivo* or cancer patient drug response and biomarkers from cell line screening data. Brief Bioinform (2021) 22(6):bbab260. doi: 10.1093/bib/bbab260 34260682 PMC8574972

[22] van Gent D Sharp P Morgan K Kalsheker N. Serpins: structure, function and molecular evolution. Int J Biochem Cell Biol (2003) 35(11):1536–47. doi: 10.1016/s1357-2725 (03)00134-1 10.1016/s1357-2725(03)00134-1 12824063

[23] Tian S Peng P Li J Deng H Zhan N Zeng Z. SERPINH1 regulates EMT and gastric cancer metastasis *via* the wnt/beta-catenin signaling pathway. Aging (Albany NY) (2020) 12(4):3574–93. doi: 10.18632/aging.102831 PMC7066881 32091407

[24] Wang Y Yan Q Mo Y Liu Y Wang Y Zhang S. Splicing factor derived circular RNA circCAMSAP1 accelerates nasopharyngeal carcinoma tumorigenesis *via* a SERPINH1/c-myc positive feedback loop. Mol Cancer (2022) 21(1):62. doi: 10.1186/s12943-022-01502-2 35227262 PMC8883650

[25] Wang Y Gu W Wen W Zhang X. SERPINH1 is a potential prognostic biomarker and correlated with immune infiltration: A pan-cancer analysis. Front Genet (2021) 12:756094. doi: 10.3389/fgene.2021.756094 35058967 PMC8764125

[26] Pan X Huang B Ma Q Ren J Liu Y Wang C. Circular RNA circ-TNPO3 inhibits clear cell renal cell carcinoma metastasis by binding to IGF2BP2 and destabilizing SERPINH1 mRNA. Clin Transl Med (2022) 12(7):e994. doi: 10.1002/ctm2.994 35876041 PMC9309750

[27] Hu H Ma J Li Z Ding Z Chen W Peng Y. CyPA interacts with SERPINH1 to promote extracellular matrix production and inhibit epithelial-mesenchymal transition of trophoblast *via* enhancing TGF-beta/Smad3 pathway in preeclampsia. Mol Cell Endocrinol (2022) 548:111614. doi: 10.1016/j.mce.2022.111614 35304192

[28] Wong CC Xu J Bian X Wu JL Kang W Qian Y. In colorectal cancer cells with mutant KRAS, SLC25A22-mediated glutaminolysis reduces DNA demethylation to increase WNT signaling, stemness, and drug resistance. Gastroenterology (2020) 159(6):2163–2180 e2166. doi: 10.1053/j.gastro.2020.08.016 32814111

[29] Berger A Paige CJ. Hemokinin-1 has substance p-like function in U-251 MG astrocytoma cells: A pharmacological and functional study. J Neuroimmunol (2005) 164(1-2):48–56. doi: 10.1016/j.jneuroim.2005.03.016 15913794

[30] Yepes S Tucker MA Koka H Xiao Y Jones K Vogt A. Using whole-exome sequencing and protein interaction networks to prioritize candidate genes for germline cutaneous melanoma susceptibility. Sci Rep (2020) 10(1):17198. doi: 10.1038/s41598-020-74293-5 33057211 PMC7560829

[31] Diaz de la Guardia-Bolivar E Barrios-Rodriguez R Zwir I Jimenez-Moleon JJ Del Val C. Identification of novel prostate cancer genes in patients stratified by Gleason classification: Role of antitumoral genes. Int J Cancer (2022) 151(2):255–64. doi: 10.1002/ijc.33988 PMC9311191 35234293

[32] Zhang C Liu X Jin S Chen Y Guo R. Ferroptosis in cancer therapy: A novel approach to reversing drug resistance. Mol Cancer (2022) 21(1):47. doi: 10.1186/s12943-022-01530-y 35151318 PMC8840702

[33] Fang Y Tian S Pan Y Li W Wang Q Tang Y. Pyroptosis: A new frontier in cancer. BioMed Pharmacother (2020) 121:109595. doi: 10.1016/j.biopha.2019.109595 31710896

[34] Wong RS. Apoptosis in cancer: from pathogenesis to treatment. J Exp Clin Cancer Res (2011) 30(1):87. doi: 10.1186/1756-9966-30-87 21943236 PMC3197541

[35] Gong Y Fan Z Luo G Yang C Huang Q Fan K. The role of necroptosis in cancer biology and therapy. Mol Cancer (2019) 18(1):100. doi: 10.1186/s12943-019-1029-8 31122251 PMC6532150

[36] Zhang H Zhang N Wu W Zhou R Li S Wang Z. Machine learning-based tumor-infiltrating immune cell-associated lncRNAs for predicting prognosis and immunotherapy response in patients with glioblastoma. Brief Bioinform (2022) 23(6):bbac386. doi: 10.1093/bib/bbac386 36136350

[37] Zhang N Zhang H Wu W Zhou R Li S Wang Z. Machine learning-based identification of tumor-infiltrating immune cell-associated lncRNAs for improving outcomes and immunotherapy responses in patients with low-grade glioma. Theranostics (2022) 12(13):5931–48. doi: 10.7150/thno.74281 PMC9373811 35966587

[38] Wu T Dai Y. Tumor microenvironment and therapeutic response. Cancer Lett (2017) 387:61–8. doi: 10.1016/j.canlet.2016.01.043 26845449

[39] Szeto GL Finley SD. Integrative approaches to cancer immunotherapy. Trends Cancer (2019) 5(7):400–10. doi: 10.1016/j.trecan.2019.05.010 PMC7467854 31311655

[40] Zhang H Wang Y Zhao Y Liu T Wang Z Zhang N. PTX3 mediates the infiltration, migration, and inflammation-resolving-polarization of macrophages in glioblastoma. CNS Neurosci Ther (2022) 28(11):1748–66. doi: 10.1111/cns.13913 PMC9532932 35855654

[41] Yin W Zhang K Deng Q Yu Q Mao Y Zhao R. AZD3759 inhibits glioma through the blockade of the epidermal growth factor receptor and janus kinase pathways. Bioengineered (2021) 12(1):8679–89. doi: 10.1080/21655979.2021.1991160 PMC8806996 34635007

[42] Lynch JT Polanska UM Delpuech O Hancox U Trinidad AG Michopoulos F. Inhibiting PI3Kbeta with AZD8186 regulates key metabolic pathways in PTEN-null tumors. Clin Cancer Res (2017) 23(24):7584–95. doi: 10.1158/1078-0432.CCR-17-0676 28972046

[43] Dasari S Tchounwou PB. Cisplatin in cancer therapy: Molecular mechanisms of action. Eur J Pharmacol (2014) 740:364–78. doi: 10.1016/j.ejphar.2014.07.025 PMC4146684 25058905

