# Supplementary material for: By using machine learning and in vitro testing, SERPINH1 functions as a novel tumorigenic and immunogenic gene and predicts immunotherapy response in osteosarcoma
Source: Front Oncol. 2023 Apr 5;13:1180191. doi: 10.3389/fonc.2023.1180191 (PMC10113657; doi:10.3389/fonc.2023.1180191)
Supplement: Supplementary file 1 [file DataSheet_1.docx]

Method

Immunohistochemistry (IHC)

Formalin-fixed paraffin-embedded osteosarcoma tissue and para-carcinoma tissue blocks from three osteosarcoma patients (post-chemotherapy) were collected and processed for 5 mm paraffin sections. IHC was performed using the Mouse/rabbit-enhanced polymer method detection system (ZSGB-BIO, PV-9000, China). The slides were deparaffinized and rehydrated using xylene and gradient-concentration ethyl alcohol. The antigen retrieval was performed with sodium citrate at 95°C. The slides were blocked using an endogenous peroxidase blocker for 10 min at room temperature. Samples were incubated with primary antibody against SERPINH1 (10875-1-AP, Proteintech, China) overnight at 4°C, reaction enhancer for 20 min at 37°C, and enhanced enzyme-conjugated sheep anti-mouse/rabbit IgG polymer for 20 min at 37°C. The slides were stained with 3, 30-diaminobenzidine tetrahydrochloride (DAB) and counterstained with hematoxylin.

Cell Culture

Two osteosarcoma cell lines (U2OS and MNNG/HOS) were obtained from the Procell Life Science&Technology Co., Ltd. U2OS and MNNG/HOS were correspondingly cultured in McCoy’s 5A (Procell, China) and MEM (Procell, China) supplemented with 10% fetal bovine serum (FBS, Gibco, USA) and 1% penicillin-streptomycin solution (Biosharp, China) at 37°C with saturated humidity and 5% CO2. The average time of culture medium exchange was 24-48h. The cells were digested with trypsin-EDTA (Gibco, USA) and passaged when cell adhesion exceeded 80% confluency.

Small interfering RNA (siRNA) transfection

The SERPINH1 siRNA (si-SERPINH1) and the nonspecific control siRNA (si-NC) were synthesized by JTSBio (Wuhan, China). The siRNAs sequences are as follows: SERPINH1-1 (F: GCAGCAAGCAGCACUACAATT R: UUGUAGUGCUGCUUGCUGCTT), SERPINH1-2 (F: CCAGCCUCAUCAUCCUCAUTT R: AUGAGGAUGAUGAGGCUGGTT), SERPINH1-3 (F: GGCCUAAGGGUGACAAGAUTT R: AUCUUGUCACCCUUAGGCCTT). The siRNAs were transfected into U2OS and MNNG/HOS cells using a jetPRIME transfection reagent (Polyplus, France). RNA extraction was performed 48h after transfection.

Real-time quantitative polymerase chain reaction (RT-qPCR)

The primer sequences are as follows: SERPINH1 (F: ATATTTATAGCCAGGTACCTTCTCACC R: TTTTATAGTTGGGAGAGGTTGGGATAG), GAPDH (F: AATGGGCAGCCGTTAGGAAA R: GCCCAATACGACCAAATCAGAG). Total RNA from cultured cells was extracted using Rnafast200 (Fastagen, Japan), and cDNA was synthesized using HiScript II Q RT SuperMix for qPCR (Vazyme, China). ChamQ Universal SYBR qPCR Master Mix (Vazyme, China) was used to conduct RT-qPCR based on the manufacturer’s protocol. All steps for the RT-qPCR reaction were performed as follows: initial denaturation at 95°C for the 30s, one cycle; denaturation at 95°C for 10s, 40 cycles; dissolution curve at 95°C for 15s, 60°C for 60s, 95°C for 15s, one cycle. Gene expression levels were normalized to those of GAPDH and calculated using lg2–△△Ct method.

Cell Counting Kit-8 (CCK-8) assay

The U2OS and MNNG/HOS cells were seeded into 96-well plates at 5,000 cells/well density. After 24h, 1/10 volume of CCK-8 reagent (Proteintech, USA) was added to the wells, and the absorbance value was detected at 450nm after 1h incubation at 37℃.

EdU assay

Proliferating U2OS and MNNG/HOS cells were identified using the Click-iT Plus EdU Alexa Fluor 488 Imaging Kit (Invitrogen, USA), and cell nuclei were stained using Hoechst (Invitrogen, USA). Image Pro-Plus version 6.0 (Media Cybernetics, USA) was used for counting EdU-positive cells.

Transwell assay

The migration of U2OS and MNNG/HOS cells was assessed using a Transwell chamber (Corning, USA) with polycarbonic membranes (6.5 mm in diameter and 8 μm pore size). Cells in serum-free medium were added into the upper chamber at the density of 5 × 10^5^ cells/ml (200 μl/well), and the culture medium with 10% FBS was added to the lower chamber. After incubating for 48h at 37°C, U2OS and MNNG/HOS cells that penetrated the lower surface were stained with 0.1% crystal violet and counted.

Supplementary Figures


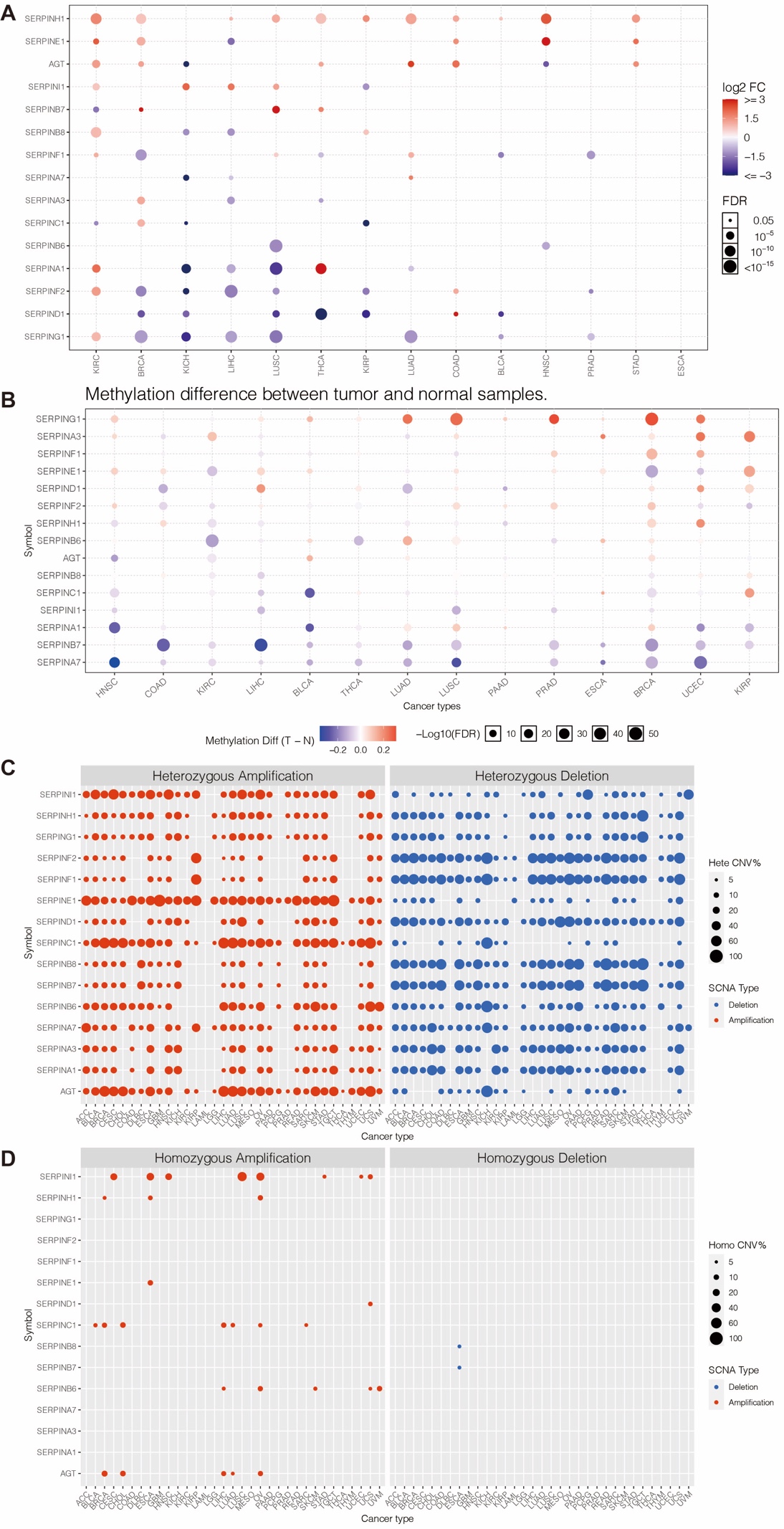


Figure S1. Pan-cancer mutation analysis on the serpin superfamily. A. The expression pattern of the serpin superfamily between tumor and normal samples. B. Methylation difference of the serpin superfamily between tumor and normal samples. C. Heterozygous CNV of the serpin superfamily. D. Homozygous CNV of the serpin superfamily.


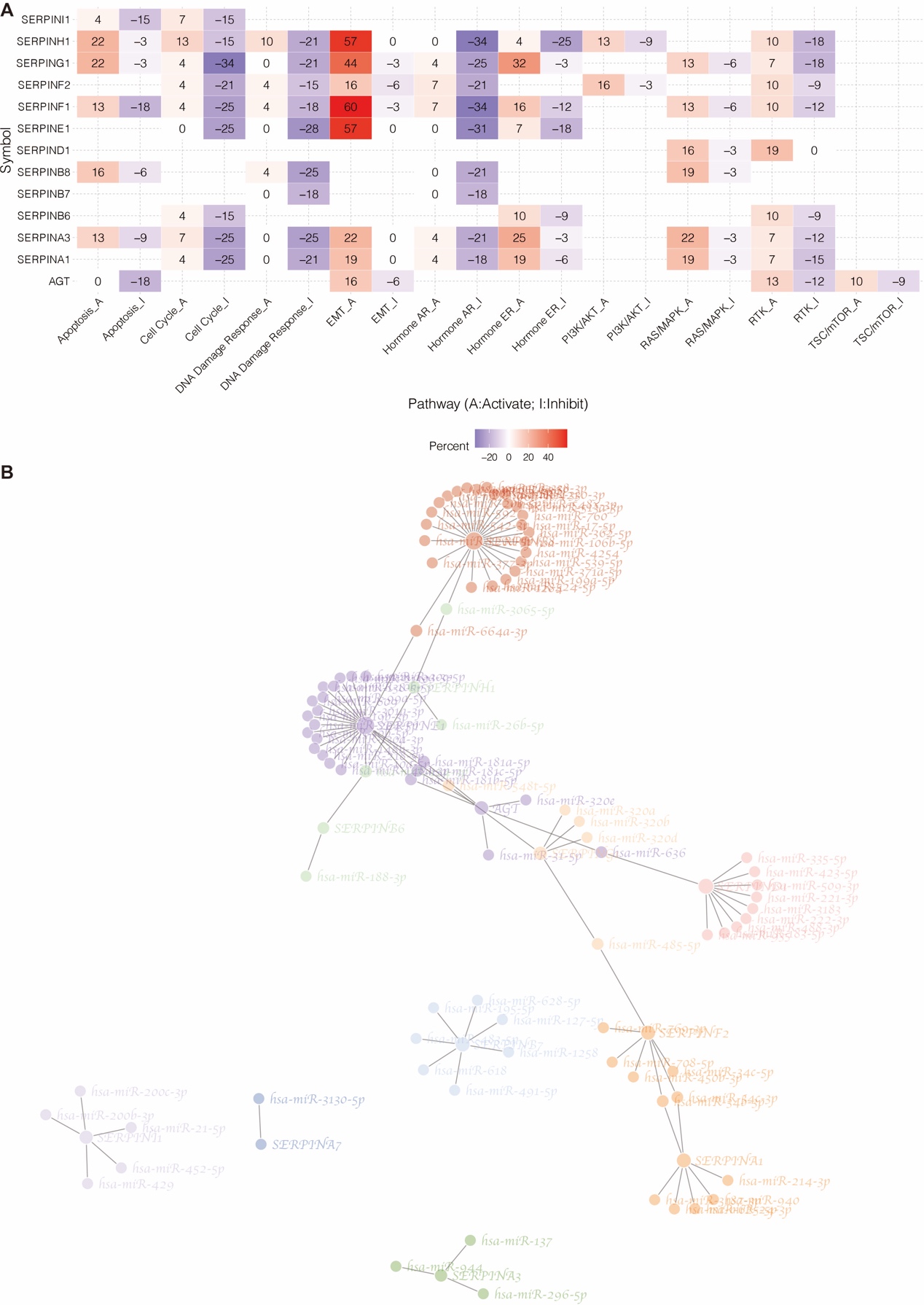


Figure S2. Pan-cancer function analysis on the serpin superfamily. A. Pathway annotation on the serpin superfamily. B. miRNA network annotation on the serpin superfamily.


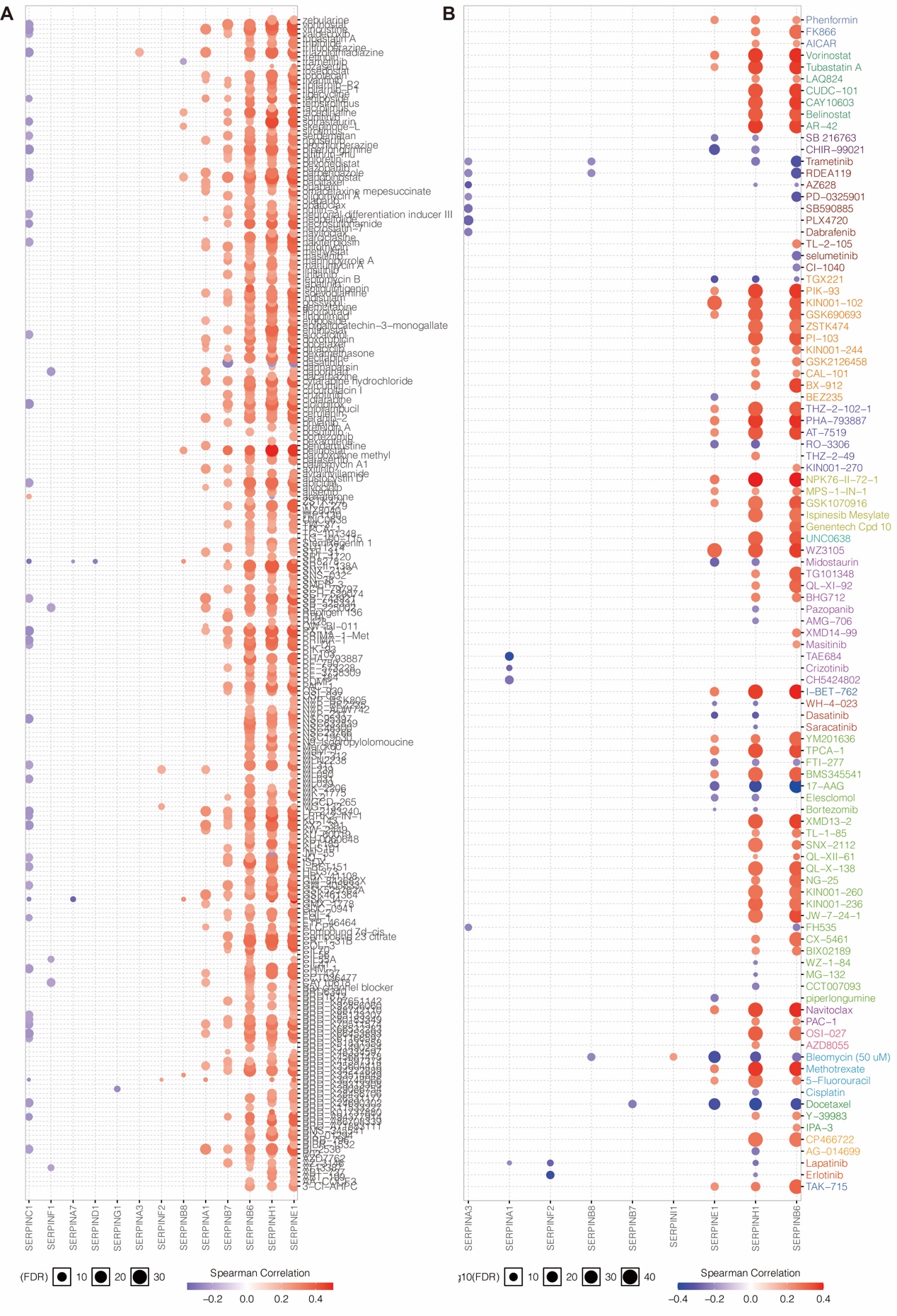


Figure S3. Pan-cancer drug prediction analysis on the serpin superfamily. A. CTRP database. B. GDSC database.


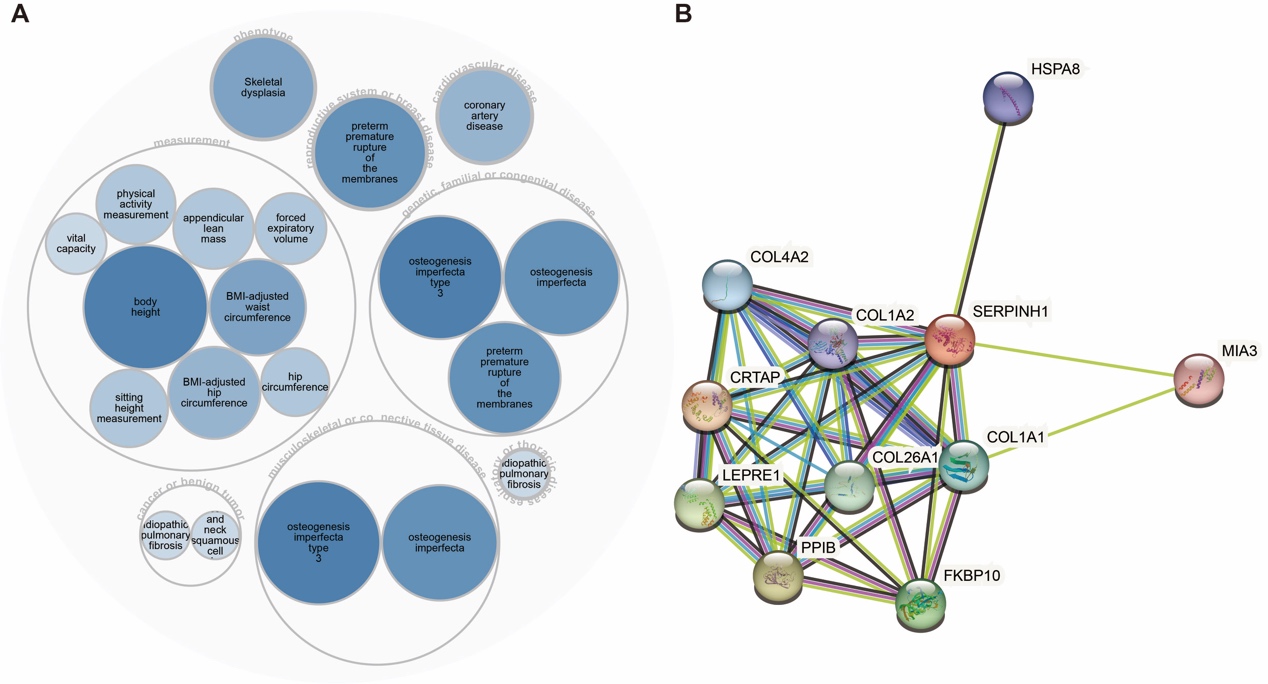


Figure S4. A. The disease network of SERPINH1. B. The protein interaction network of SERPINH1.
